# Hypoxia-reoxygenation induced necroptosis in cultured rat renal tubular epithelial cell line

**DOI:** 10.22038/IJBMS.2018.26276.6444

**Published:** 2018-08

**Authors:** Changlai Zhu, Yang Liu, Zongyu Guan, Yi Zhou, Fang Liu, Tianyi Zhang

**Affiliations:** 1 Jiangsu Key Laboratory of Neuroregeneration, Co-innovation Center of Neuroregeneration, Nantong University, Nantong, JS 226001, P. R. China; 2 Medical College of Nantong University, Nantong, JS, P. R. China

**Keywords:** Cell line, Hypoxia-Reoxygenation Nec-1, Necrosome, Receptor interacting protein

## Abstract

**Objective(s):**

The aim of this study is to explore the potential role of hypoxia/reoxygenation in necroptosis in cultured rat renal tubular epithelial cell line NRK-52E, and further to investigate its possible mechanisms.

**Materials and Methods:**

Cells were cultured under different hypoxia-reoxygenation conditions *in vitro*. MTT assay was used to measure the cell proliferation of cells that were exposed to hypoxia-reoxygenation conditions at different time points. Receptor-interacting protein 1,3 (RIP1 and RIP3) and NF-κB were detected by Western-blot analysis. Co-immunoprecipitation (Co-IP) was conducted to investigate the formation of necrosome. Necrostatin-1 (Nec-1) was adopted to inhibit the occurrence of necroptosis. In addition, morphological changes of cells after hypoxia-reoxygenation interference were observed under transmission electron microscope (TEM).

**Results:**

MTT assay indicated that hypoxia-reoxygenation treatment can cause a decrease in cell viability. Particularly, 6 hr of hypoxia and 24 hr of reoxygenation (H6R24 group) resulted in the lowest cell viability. Western-blot results indicated that the expression of RIP3 significantly increased in H6R24 group while the expression of NF-κB is decreased. Co-IP results demonstrated that the interaction between RIP1 and RIP3 was stronger in the hypoxia-reoxygenation induced group than the other groups, furthermore, treatment with Nec-1 reduced the formation of necrosome. TEM observation results showed that hypoxia-reoxygenation treated cells showed typical morphological characteristics of necroptosis and autophagy.

**Conclusion:**

Hypoxia-reoxygenation treatment can induce necroptosis in NRK-52E cells, and this effect can be inhibited by Nec-1. In addition, the mechanism of necroptosis induced by hypoxia-reoxygenation injury on cells may be related to the low expression of NF-κB.

## Introduction

Traditionally, cell death has been categorized into programmed cell death (mainly due to apoptosis) and necrosis (1, 2). However, a growing number of researchers found that necroptosis, distinct from apoptosis and necrosis, contributes to a range of physiological or pathological processes as a new mechanism of cell death, and also closely correlates to some necrotic diseases in their occurrence, development, as well as the final outcome (3-6). Necroptosis has attracted much more research interests in recent years.

Receptor-interacting kinase 1 (RIP1) was considered to be an important regulatory factor of necrosis in caspase-inhibited cells (7), and researchers had also found that small-molecule drugs can inhibit this kind of programmed cell death (8). Subsequently, this inhibitory drug of cellular programmed necrosis is shown to be the target on the RIP1 molecule (9). Also, researchers have confirmed that the receptor interaction proteins 3 (RIP3) is the downstream mediator of RIP1 (10, 11). Many *in vivo* studies also suggested that RIP1, RIP3, and MLKL (Mixed lineage kinase domain-like protein) are the main participants in the execution of such cell death (12). Many studies also indicated that many tumor cells, which fail to die by apoptosis in the body, can be killed by triggering the necroptosis procedure, suggesting that this kind of safeguard mechanism is vital to cancer biology and therapy. But the role of necroptosis on human ischemia-reperfusion (I/R) injury is not widely reported.

The kidneys are very easily influenced by I/R injury due to their special physical structure and function. Renal I/R has become a common clinical problem associated with acute kidney injury (AKI), which may progress to chronic kidney disease and lead to a high mortality finally. Its typical clinical features mainly include severe decrease in glomerular filtration rate, a significant decline in renal blood flow, as well as the renal tubular epithelial cell injury (13, 14). Although many studies have demonstrated that the generation of reactive oxygen species (ROS), the accumulation of toxic substances in kidney cells, and release of cytokine synergistically, play important roles in the pathophysiological mechanism of AKI (15, 16), the detailed molecular mechanisms underlying I/R injury are incompletely understood up to now. Since renal tubular injury caused by ischemia-hypoxia is a very common clinical problem, it’s very essential to investigate the detailed pathological and pathophysiological mechanisms, which can provide the theoretical support and new prospects for further clinical treatment.

Therefore, investigation of the potential molecular mechanisms, relevant signaling pathways, and clinical relevance of necroptosis may be beneficial to provide useful help for molecular target therapy of AKI caused by renal I/R (2).

## Materials and Methods

***Cell culture and*** ***experimental grouping***

Rat renal tubular epithelial cell line (NRK-52E) was purchased from ATCC (American Type Culture Collection). Cells were grouped by hypoxia-reoxygenation group (HR) and normal control group.

Cell culture was performed in DMEM culture medium (Gibco, America) containing 10% fetal bovine serum (FBS, Gibco). When the confluence reached 80%, cells were transferred to a hypoxia-reoxygenation workstation and exposed to anoxia environment (94% N_2_, 5% CO_2_, 1% O_2_ and with saturated humidity at 37 °C) for 2 hr, 4 hr, 6 hr, 8 hr, and 12 hr (recorded as H2, H4, H6, H8, and H12, respectively); then cells were reoxygenated for 1 hr, 12 hr, and 24 hr (recorded as R1, R12, and R24, respectively). 10 µM Nec-1 was used to inhibit the hypoxia-reoxygenation induced necroptosis. Control group cells at each time point were cultured in a conventional cell culture incubator.

**MTT assay**

MTT assay was performed in 96-well plates. Briefly, the main steps are as follows: the MTT solution was added to a final concentration of 0.5% when the cells were cultured to the specified time point. Cultures were returned to the incubator for 4 hr and the culture fluid was then removed, followed by adding 150 µl DMSO to each well. Subsequently, the absorbance value at a wavelength of 570 nm was measured in a spectrophotometer.

***Western blot analysis***

The western blotting analysis was conducted as previously reported (17). Briefly, the samples were boiled for 5 min and separated by SDS PAGE at polyacrylamide gels; transferred on to PVDF membranes, and incubated with the primary antibody (mouse anti RIP1 IgG, mouse anti RIP3 IgG) (Cell Signaling Technology, America) and rabbit anti NF-κB p65 antibody (Sigma) at 4 ^°^C for 12 hr, rinsed twice with 10 mM phosphate buffer solution, and incubated with the horseradish peroxidase-conjugated secondary antibody for 2 hr at room temperature. After three times washing, the samples were visualized by enhanced chemiluminescent (ECL) reagents.

***Co-Immunoprecipitation analysis***

Co-Immunoprecipitation detection was performed according to a previous report (18). Briefly, cells were harvested and the whole cell lysates were prepared. A total of 500 µg of protein extract was then incubated with 1 µg of the appropriate immunoprecipitation (IP) antibody for 1 hr under rotation. Then, 20 µl of protein G Sepharose (Sigma–Aldrich) was added, followed by incubation at 4 ^°^C overnight under rotation. The pellets were collected and washed four times with cell lysis buffer for 5 min. The pellets were re-suspended in 40 µl of 2×electrophoresis loading buffer boiled for 5 min. Then, 20 µl samples were used for gel electrophoresis, and the subsequent steps were the same as the Western-blot analysis.

***Electron microscopy***

Cells were washed twice with 10 mM phosphate buffer solution (PBS) and fixed with 2.5% glutaraldehyde for 2 hr, washed three times with PBS and then post-fixed with 1% osmium tetroxide, dehydrated with ethanol, followed by embedding in epoxy resin. Ultrathin sections were then double-stained with 1% lead citrate and 0.5% uranyl acetate. Image observation and acquisition were conducted under an electron microscope (HT-7700, Hitachi, Japan) at 80 kV.

***Statistical analysis***

Data are expressed as the mean ± SD and were analyzed by Student’s *t*-test.

## Results

***Cell viability detection***

**Figure 1:**
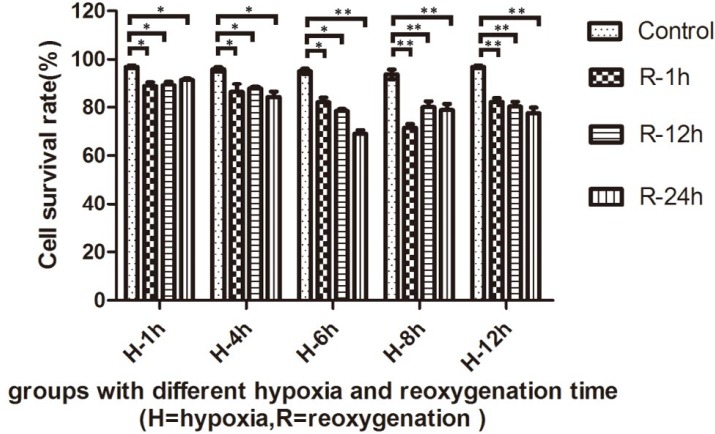
The anti-proliferative effect of the hypoxia-reoxygenation intervention in NRK-52E cells. Cells were exposed to the indicated hypoxia-reoxygenation time. Cell survival rate was detected using MTT assay. (** *P*<0.05)

To study the cell inhibition effect of different time periods of hypoxia-reoxygenation exposure, we examined the cytotoxic effects of serial time points (2 hr, 4 hr, 6 hr, 8 hr, and 12 hr) of hypoxia and reoxygenation (1 hr, 12 hr, and 24 hr) environment using the MTT assay. The results showed that Hypoxia- reoxygenation intervention significantly inhibited cell growth in a time-dependent manner (Figure 1). The viability of cells, which were treated with hypoxia-reoxygenation intervention, had an obvious decrease (*P*<0.05) as compared to the control groups. Moreover, cell growth was inhibited to approximately 68% and 75% in H6R24 and H8R1 groups, respectively. The results showed that the survival rate of NRK-52E cells was lowest in 6 hr of hypoxia and 24 hr of reoxygenation.

***RIP1 expression after different hypoxia-reoxygenation induction times***

**Figure 2:**
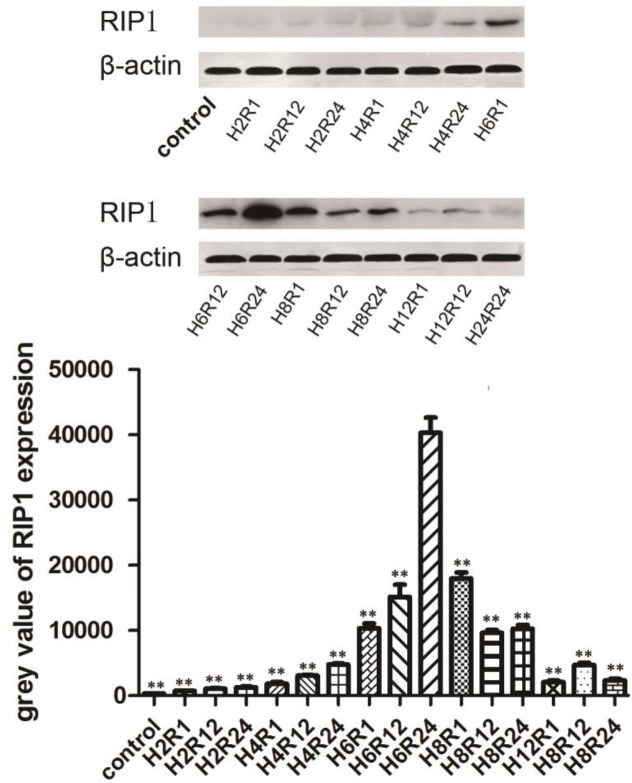
The grey value of RIP1 expression level in different times of exposure to hypoxia-reoxygenation. Grey value of H6R24 group was significantly higher than in other groups. The quantitative analysis results were shown as mean grey value±SD (** *P*<0.05)

To investigate the relationship between RIP1 expression and the variations of the time of hypoxia-reoxygenation, Western blot analysis was carried out. The results confirmed that the level of RIP1 was closely related to the time of the hypoxia-reoxygenation. In the blank control group, 1 hr, 12 hr, and 24 hr of reoxygenation after 2 hr of hypoxia groups, there was no obvious RIP1 expression. After 4 hr of hypoxia, there was a low level of RIP1 expression with the increase of the time of reoxygenation. In three reoxygenation groups after 6 hr of hypoxia, however, there was an obvious increase of the RIP1 level, which was remarkably high in the H624 group. Subsequently, the RIP1 level was gradually decreased with the time of hypoxia-reoxygenation increasing (Figure 2).

***Necrosome detection***

**Figure 3:**
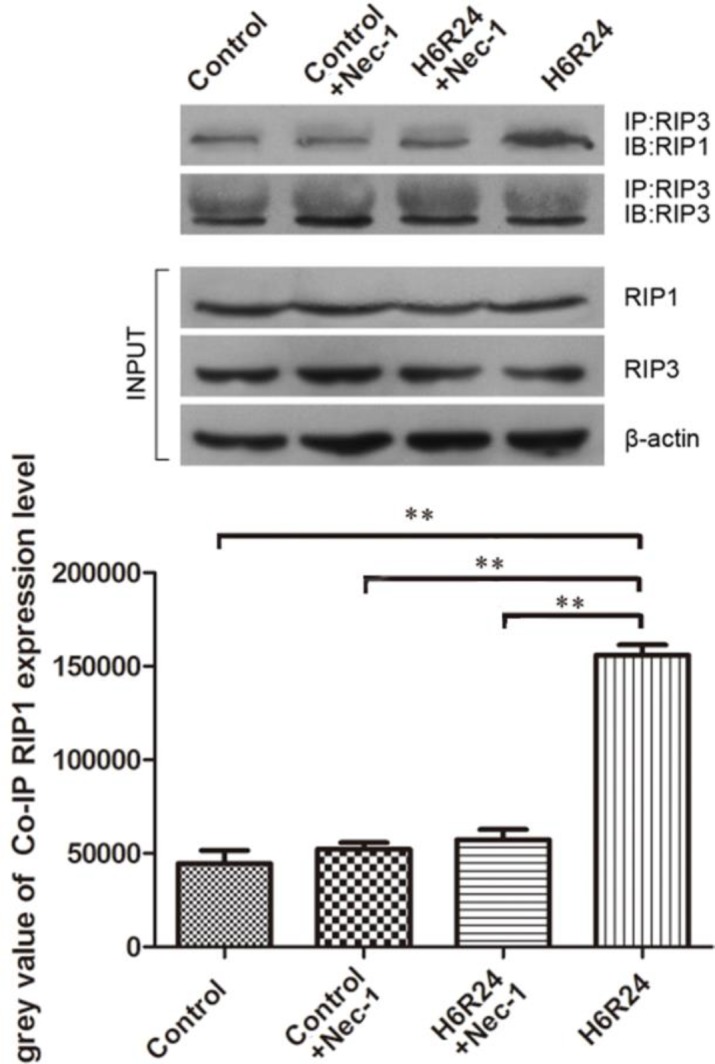
The Co-immunoprecipitation assay was analyzed by the Western-blot method. Whole cell lysates were immunoprecipitated with anti-RIP3, and the Co-immunoprecipitated complexes were immunoblotted for RIP1 and RIP3 (IP=immunoprecipitation, IB=immunoblot). Quantitative analysis of RIP1 expression level in different groups was also shown (** *P*<0.05)

Necrosome detection by Co-immunoprecipitation and Western-blot analysis Western-blot results demonstrated that the 6 hr of hypoxia-reoxygenation groups showed the obvious RIP1 expression, suggesting that there was presence of necroptosis. Therefore, co-immunoprecipitation was performed to further detect the formation of necrosome, which consisted of RIP1 and RIP3 complexes, the specific markers of necroptosis. The RIP1 and RIP3 antibodies were used as capture (IP) and detector (IB) molecules, respectively, The results indicated that in three 6 hr of hypoxia-reoxygenation groups, there were obvious targeted capture and detector protein expressions, while they were not detected in the control group (Figure 3).

***Necrosome detection after Nec-1 interference***

**Figure 4:**
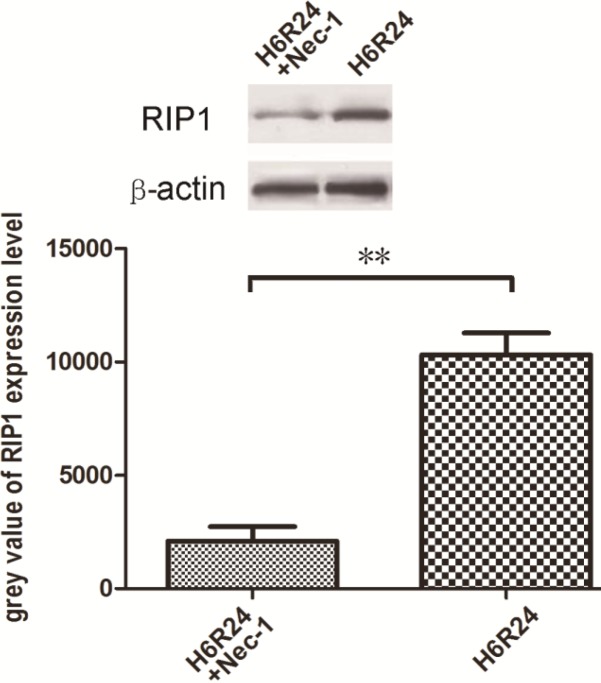
Western-Blotting analysis of RIP1 expression level in H6R24 and H6R24+Nec-1 groups. The quantitative analysis results were expressed as mean grey value±SD (** *P*<0.05)

Nec-1 is the allosteric effector of RIP1 kinase and we subsequently applied it to prevent the hypoxia-reoxygenation-induced necroptosis and to further detect the necrosome. In the H6R24 group (6 hr of hypoxia and 24 hr of reoxygenation), when 10 mM of Nec-1 was added to the culture medium for inhibition of necroptosis, the RIP1 protein expression was significantly lower than in the negative control group (*P*<0.05); and no co-immunoprecipitated molecular could be detected after the interference of Nec-1 (Figure 4).

***NF-*** ***κ*** ***B level after hypoxia-reoxygenation induction***

**Figure 5:**
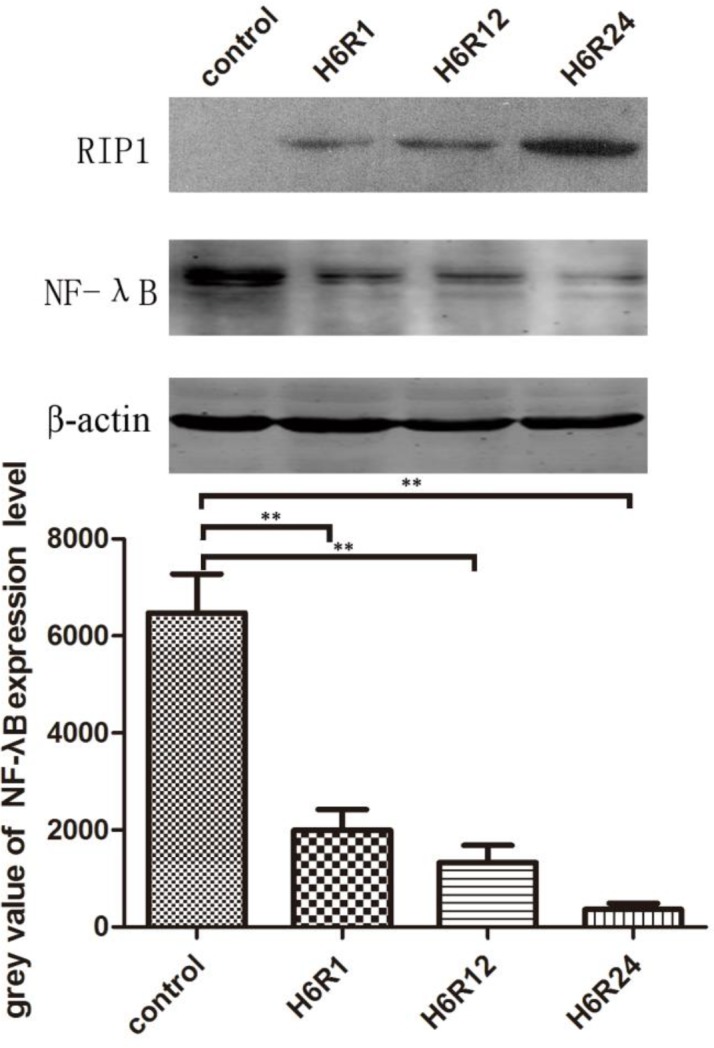
NRK-52E cells were exposed to various reoxygenation times after 6 hr of hypoxia, and then NF-κB, RIP1 expression was detected by the Western-blot method

As a regulator of genes, NF-κB plays a primary role in regulating cell survival and cell proliferation in eukaryotic cells. Here, we detected the NF-κB expression level after 6 hr of hypoxia and three time points of reoxygenation to further evaluate the potential mechanism. The results implied that there was a  negative correlation between the NF-κB expression level and necrosome; they showed that in the control group, in which the cells were in normal culture state without any intervention, the NF-κB expression was significantly higher than in the three hypoxia-reoxygenation intervention groups (*P*<0.05) (Figure 5).

***TEM observation***

**Figure 6:**
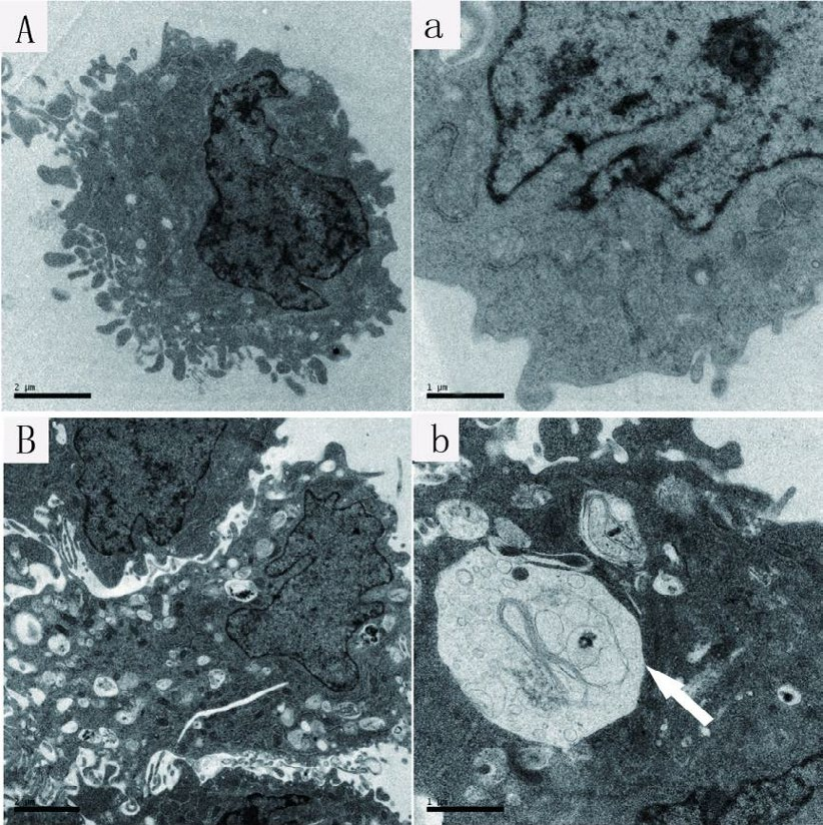
Ultrastructure features of NRK-52E cells in the control group (A, a) and H6R24 group (B, b). Scale bars, as indicated in the images, white arrowhead shows characteristic autophagosome

The ultra-structural information of hypoxia-reoxygenation induced cells (H6R24 group) and untreated cells (control group) were examined using a TEM. Cells in the control group showed the intact plasma membrane with no obvious necroptotic and autophagic features (Figures 6A and 6a). In hypoxia-reoxygenation induction (H6R24) group, cells exhibited cytoplasm swelling and vacuolization; and autophagosome also can be observed in cytoplasms (Figures 6B and 6b).

## Discussion

Due to the lack of an effective treatment strategy, renal ischemia-reperfusion injury often causes a serious consequence including severe reduction in glomerular filtration rate and renal tubular epithelial cell injury, it’s essential to investigate the detailed pathological and pathophysiological mechanisms of this injury. In this study, *in vitro* culture of NRK-52E cells was carried out in hypoxia/reoxygenation condition to simulate the *in vivo* renal ischemia-reperfusion injury process caused by AKI.

More recently, necroptosis, a nonapoptotic pathway of cell death, has been described and widely provoked concern. Many researchers have reported that renal I/R injury will cause the apoptosis and necrosis of renal tubular epithelial cells, but there seem to be no studies that have explored the relationship between the necroptosis and renal I/R caused AKI. In the present study, therefore, we establish the *in vitro* cell culture model to simulate the I/R injury *in vivo* and to investigate the role and mechanism of necroptosis in the development of I/R injury.

MTT assay results showed that the survival rates of cells at 2 hr and 4 hr of hypoxia groups were only slightly lower than those of the normal control group; these results indicate that the damage to the cells that underwent a short period of hypoxia can still grow well after reoxygenation. In 6 hr of hypoxia groups, however, the survival rates were significantly decreased after reoxygenation. The cell survival rate in the H6R24 group was the lowest, suggesting that reoxygenation is the leading cause of cell hypoxia-reoxygenation injury, and 6 hr of hypoxia probably induced the high expression of hypoxia-inducible factor 1 (HIF-1), which increases the tolerance of cells to hypoxia (19). In 8 hr and 12 hr hypoxia groups, however, the survival rate of cells decreased obviously after hypoxic treatment, but it did not further decline after the reoxygenation interference. These results further implied that in 8 hr and 12 hr of hypoxia/reoxygenation groups, the injury to the cells is mainly related to hypoxia, the reason may be declined cell tolerance to a long time of hypoxia, and its damage to cells is more than reoxygenation. Therefore, we selected the 6 hr of hypoxia and reoxygenation groups for further research. 

The mechanisms of necroptosis have been studied very extensively by many researchers (20, 21) and the key role of RIP1 kinase in the necroptotic process is firmly established. Previous reports have demonstrated that RIP1 kinase is vital for activating NF-κB and generation of ROS. In addition, RIP1 is also involved in activation of MAPKs under certain conditions, such as p38 MAPK, JNK, and ERK (22). Moreover, its activity was shown to be critical for caspase-independent DR-mediated death (8, 9) when a death receptor is triggered. As a regulated form of programmed necrosis, it is defined by its dependence on early activation of RIP1 and the fact that it is initiated by formation of necrosomes. RIP1 is recruited to the signaling complex of death receptors and is polyubiquitinated. Therefore, to get more insights into the related molecular variation of cell death after hypoxia-reoxygenation induction in rat renal tubular epithelial cells, we have investigated the role of RIP1 by Co-immunoprecipitation technology. In the current study, we found that there were characteristic necrosome formation and higher expression levels of the RIP1 protein in NRK-52E cells in the H6R24 group. However, co-treatment with Nec-1 resulted in a significant reduction of the necrosome formation, and RIP1 expression level can be inhibited by 10 mM Nec-1, suggesting that necroptosis plays an important role in the development of cell injury caused by hypoxia/reoxygenation injury. As is well known, Nec-1 targets the RIP1 kinase step in the necroptosis pathway, while RIP1 is a crucial molecule for signaling to NF-κB, MAPKs, and ROS (22, 23). 

NF-κB is very important to eukaryotic cells in order to control cell proliferation and cell survival as a regulator of genes. When the NF-κB pathway is being activated, the transcription factor subunits (mainly refers to the p65 and p50) bind to the regulatory elements of DNA to regulate the gene transcription. In addition, the vital role of NF- κB also involves regulating immune and inflammatory responses and activating cell survival programs to respond to certain cell injuries such as those induced by TNF-α (24, 25). Thapa *et. al.* have also demonstrated that mammalian cells deficient in NF-κB signaling are susceptible to IFN--γ induced RIP1-dependent necroptosis (26). Based on the above-mentioned considerations, we evaluated the NF-κB activity by analyzing translocated p65 in the nuclear fraction. Our results also indicated that when the hypoxia/reoxygenation interventions were extended to 12 hr, the expression of NF-κB p65 could be significantly decreased by Nec-1 (Figure 5). This inhibitory effect of Dec-1 further confirmed that the kinase activity of RIP1 is essential for the activation of NF-κB (27, 28). Our results also implied that the inhibited NF-κB expression may be one of the reasons for hypoxia-reoxygenation-induced necroptosis. 

Pathways leading to necroptosis are various. A new emerging hypothesis is that mitophagy, the autophagy-dependent elimination of mitochondria, also contributes to necroptosis. During starvation, cells can sustain viability by inhibiting autophagic degradation of mitochondria through mitochondrial elongation (29). Our results also indicated that autophagy is also involved in the process of apoptotic necrosis causing by hypoxia and reoxygenation, suggesting that autophagy is involved in and contributes to the pathological and pathophysiological processes of necroptosis induced by hypoxia and reoxygenation. 

## Conclusion

In the current study, we found that rat renal tubular epithelial cells could effectively induce necroptosis under hypoxia-reoxygenation conditions, and this necroptosis can be blocked by Nec-1. Further investigation indicated that the low expression of NF-κB and increase in autophagy are probably the important mechanisms of necroptosis induced by reoxygenation injury of NRK-52E cells. The results may be beneficial to understanding the detailed mechanisms of AKI caused by renal I/R and provide new prospects for further molecular target therapy.

## Conflicts of Interest

No benefits in any form have been received or will be received from a commercial party related directly or indirectly to the subject of this article.
